# Wound drainage after arthroplasty and prediction of acute prosthetic joint infection: prospective data from a multicentre cohort study using a telemonitoring app

**DOI:** 10.5194/jbji-8-59-2023

**Published:** 2023-02-13

**Authors:** Henk Scheper, Rachid Mahdad, Brenda Elzer, Claudia Löwik, Wierd Zijlstra, Taco Gosens, Joris C. T. van der Lugt, Robert J. P. van der Wal, Rudolf W. Poolman, Matthijs P. Somford, Paul C. Jutte, Pieter K. Bos, Richard E. Zwaan, Rob G. H. H. Nelissen, Leo G. Visser, Mark G. J. de Boer, the wound care app study group

**Affiliations:** 1 Department of Infectious Diseases, Leiden University Medical Centre, Leiden, the Netherlands; 2 Department of Orthopaedic Surgery, Alrijne Hospital, Leiderdorp, the Netherlands; 3 Department of Orthopaedic Surgery, University Medical Centre Groningen, Groningen, the Netherlands; 4 Department of Orthopaedic Surgery, Medical Centre Leeuwarden, Leeuwarden, the Netherlands; 5 Department of Orthopaedic Surgery, Elisabeth-TweeSteden Hospital, Tilburg, the Netherlands; 6 Department of Orthopaedic Surgery, Reinier Haga Orthopaedic Centre, Zoetermeer, the Netherlands; 7 Department of Orthopaedics, Leiden University Medical Centre, Leiden, the Netherlands; 8 Department of Orthopaedic Surgery, Onze Lieve Vrouwe Gasthuis, Amsterdam, the Netherlands; 9 Department of Orthopaedic Surgery, Rijnstate Hospital, Arnhem, the Netherlands; 10 Department of Orthopaedics and Sports Medicine, Erasmus MC, University Medical Centre Rotterdam, Rotterdam, the Netherlands; 11 Department of Biomedical Data Sciences, Advanced Data Management, Leiden University Medical Centre, Leiden, the Netherlands; ➕ A full list of authors appears at the end of the paper

## Abstract

**Background**: Differentiation between uncomplicated and complicated postoperative wound drainage after arthroplasty is crucial to prevent unnecessary reoperation. Prospective data about the duration and amount of postoperative wound drainage in patients with and without prosthetic joint infection (PJI) are currently absent. **Methods**: A multicentre cohort study was conducted to assess the duration and amount of wound drainage in patients after arthroplasty. During 30 postoperative days after arthroplasty, patients recorded their wound status in a previously developed wound care app and graded the amount of wound drainage on a 5-point scale. Data about PJI in the follow-up period were extracted from the patient files. **Results**: Of the 1019 included patients, 16 patients (1.6 %) developed a PJI. Minor wound drainage decreased from the first to the fourth postoperative week from 50 % to 3 %. Both moderate to severe wound drainage in the third week and newly developed wound drainage in the second week after a week without drainage were strongly associated with PJI (odds ratio (OR) 103.23, 95 % confidence interval (CI) 26.08 to 408.57, OR 80.71, 95 % CI 9.12 to 714.52, respectively). The positive predictive value (PPV) for PJI was 83 % for moderate to heavy wound drainage in the third week. **Conclusion**: Moderate to heavy wound drainage and persistent wound drainage were strongly associated with PJI. The PPV of wound drainage for PJI was high for moderate to heavy drainage in the third week but was low for drainage in the first week. Therefore, additional parameters are needed to guide the decision to reoperate on patients for suspected acute PJI.

## 1 Introduction

Total hip and knee arthroplasties are highly successful treatment modalities for advanced osteoarthritis, the most common joint disorder worldwide (Hiligsmann et al., 2013). A prosthetic joint infection (PJI), which develops in approximately 1 %–2 % of all arthroplasties, is a serious and devastating postoperative complication with a high impact on a patient's well-being (Kurtz et al., 2012; Zimmerli et al., 2004). Postoperative wound drainage is frequently reported as an important indicator for the presence of PJI (Wagenaar et al., 2019; Patel et al., 2007; Almeida et al., 2021). Wound drainage may be an early symptom of a present PJI but may also be a risk factor for the subsequent development of PJI (Patel et al., 2007; Weiss and Krackow, 1993). Discrimination between infectious and non-infectious postoperative wound drainage is of crucial importance. When the prosthetic joint is infected, surgical debridement and protracted antimicrobial treatment is required. For noninfectious serosanguinous drainage caused by intraoperative disruption of soft tissue and capillaries, only conservative wound management is indicated (Wagenaar et al., 2019).

In 2013, the first International Consensus Meeting (ICM) on PJI advised that surgical management of persistent wound drainage should be performed without delay if wound drainage persists for 5–7 d after index surgery (Parvizi et al., 2013). According to the recently published EBJIS (European Bone and Joint Infection Society) definition for PJI, a history of prolonged wound drainage (as a feature of wound healing problem) is a clinical sign included in the PJI likely category (Mcnally et al., 2021). However, these recommendations were not backed up by research data about duration of postoperative wound drainage as summarized in a recent systematic review (Wagenaar et al., 2019). Collecting wound drainage data is challenging because most patients are discharged from hospital soon after surgery. The use of smartphone applications (apps) for distant telemonitoring of postoperative patients has proven to be feasible and acceptable for both patients and surgeons (Sanger et al., 2014; Gray et al., 2010; Chua et al., 2017; Armstrong et al., 2017). In an earlier study, the use of a postoperative wound care app that was developed at the Leiden University Medical Centre showed a high perceived usefulness and ease of use as reported by patients (Scheper et al., 2019). To assess the amount and duration of postoperative wound drainage after joint arthroplasty in patients with and without PJI, we conducted a nationwide cohort study using this smartphone application in which we collected detailed information regarding the condition and natural history of the postoperative wound.

## 2 Methods

A multicentre, prospective observational study was conducted in 11 Dutch academic and non-academic hospitals between 1 November 2019 and 1 October 2021. All patients aged 18 years and older who received a knee or hip arthroplasty, who were able to provide informed consent, owned an Android or iOS smartphone and were able to read the Dutch language, were eligible for inclusion. Patients were screened during or after preoperative visits by a local nurse specialist or the coordinating study nurse. Informed consent was obtained via the wound care app. Instructions on how to use the app were provided to all patients by the local research coordinator. The nurses in the ward as well as the study coordinator were available for help with the use of the app during admission and throughout the study. All patients received routine postoperative medical care in the outpatient clinic as per local protocol in each participating hospital. The primary endpoint was the extent and duration of the postoperative wound drainage in patients with and without PJI. Secondary endpoints were the association between the presence of self-reported fever, redness, pain and PJI, and the validation of the designed algorithm for sending alert messages for suspected PJI. The PJI was defined according to the criteria from the European Bone and Joint Infection Society (EBJIS; Mcnally et al., 2021).

The use and function of the app has been described previously (Scheper et al., 2019). In short, for 30 d following joint arthroplasty, patients recorded their wound status daily on their mobile app. Redness, pain (by visual analogue score, VAS), wound drainage and presence of fever were recorded, and a picture of the wound could be taken. Based on the questionnaires, an inbuilt algorithm created a daily risk score (see Appendix A). If this score exceeded a predefined threshold, which was based on expert consensus of participating clinicians, an alert message was issued that allowed patients to contact their treating physician via a push button in the app. It was for the attending clinicians to decide whether patients needed a clinical review or not. If wound drainage during the first 14 d was not reported, patients were allowed to stop using the app. They were instructed to resume the use of the app if new drainage or other complications arose. After both 30 and 90 d, all patients were asked to report postoperative complications in the app. After a minimum follow-up period of 90 postoperative days, endpoint data were extracted from both the app and the electronic patient files to enable a comparison of the patient-reported and physician-reported outcome. If discordant, the outcome reported by the attending orthopaedic surgeon was regarded as the final outcome.

The study was conducted according to the principles of the Declaration of Helsinki. The study was approved by the ethics review committees and a waiver was obtained to use electronic instead of written informed consent. The use of the app for this study was approved by the Dutch Health Inspectorate (reference number VGR2O1 1434). The app was developed by the software company Innovattic. This company was not involved in the setup, data analysis and report of this study.

**Table 1:** Self-reported wound characteristics by patients in the wound care app.

Characteristic	Daily available scores for the patient after surgery
Fever	T<38 ${}^{\circ }$ C
	T38 –38.5 ${}^{\circ }$ C
	T>38.5 ${}^{\circ }$ C
Wound drainage	No
	Minimal: <2×2 cm on bandage
	Mild: >2×2 cm on bandage
	Moderate: 1–2 bandages exchanged
	Heavy: >2 bandages exchanged
	Not judgeable (e.g. due to plaster or wound dressing)
Redness of wound	No
	Yes, less red than yesterday
	Yes, same as yesterday
	Yes, increased compared to yesterday
	Not judgeable (e.g. due to plaster or wound dressing)
Pain score (visual analogue score)	Score 0–10 (via a slider in the app)

**Figure 1:**
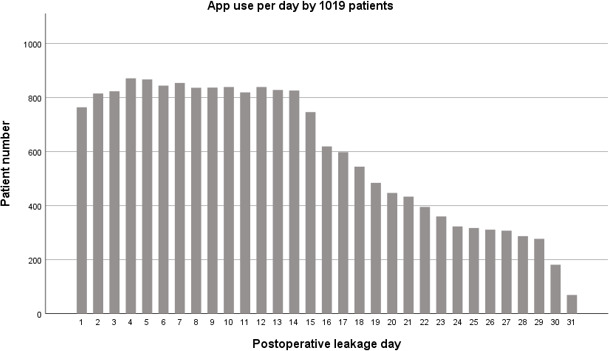
Daily wound care app use by patients during postoperative period.

### 2.1 Quantification of wound drainage

The International Consensus Meeting (ICM) on PJI defined persistent wound drainage as >2×2 cm of drainage in the wound dressing beyond 72 h after index surgery. However, this definition lacks a more detailed quantification of wound drainage (Parvizi et al., 2013). Therefore, we used a proposed classification of persistent wound drainage which is currently used in another Dutch wound drainage study (National Trial Registration 5960) (Lowik et al., 2017). On a daily basis, the patient had to enter the following drainage scores into the app: no drainage, minimal drainage, mild drainage, moderate drainage or heavy drainage (for exact definitions, see Table 1).

**Table 2:** Baseline and outcome characteristics of 1019 patients as entered into the app ${}^{a}$ .

	Reported by patient in app	Definite report by study team
Baseline characteristics		
Age (median, range)	65 (18–90)	n/a
BMI (mean, SD)	29.1 (11.0)	n/a
Type of joint arthroplasty		
Knee	467 (46 %)	n/a
Hip	547 (54 %)	n/a
Other (shoulder, ankle)	2 (0.2 %)	n/a
Tumour prosthesis ( n , %)	10 (1 %)	n/a
Past medical history		
Diabetes mellitus	73 (7 %)	n/a
Rheumatoid arthritis	60 (6 %)	n/a
Report of outcome		
Prosthetic joint infection (PJI)	16 (1.5 %)	16 (1.6 %)
Surgery for suspected PJI, appeared to be no PJI	5 (0.5 %)	3 (0.3)
Superficial wound infection, resolved after antibiotic treatment	5 (0.5 %)	6 (0.6 %)
Superficial wound infection, spontaneously resolved	22 (2.2 %)	2 (0.2 %)
No data available	176 (17.5 %) ${}^{b}$	39 (3.8 %) ${}^{c}$
I do not know	121 (11.9)	–
No complication (if data available)	674/843 (80.0 %)	956/980 (97.6 %)

${}^{a}$ Outcome checked until 3 months post operation; ${}^{b}$ 179 patients did not fill out the outcome after 30 and 90 d. ${}^{c}$ From one study centre, data from the 39 included patients could not be retrieved from the local researcher. n/a – not applicable.

### 2.2 Statistical analysis

Descriptive statistics were used for baseline characteristics. To address missing values of wound drainage, the most recent drainage score was carried forward if data were missing after the first 14 d but only if the most recent drainage score was “no drainage”. The cut-off of 14 d was based on the app recommendation to stop using it after 14 d and only resume use if any new complications arose. Odds ratios (ORs), sensitivity, specificity, and negative and positive predictive values (NPVs and PPVs) were calculated to examine the strength of the association between mild or moderate to heavy wound drainage and PJI and between the duration of wound drainage and PJI. Median duration of wound drainage was compared between patients with and without PJI using Mann–Whitney U test. All statistical analyses were performed with SPSS (IBM SPSS Statistics version 25.0, Armonk, USA).

### 2.3 Data flow and management

Privacy-sensitive data entered into the app by patients were pseudonymized with trusted real-time encryption. Encryption keys and a list of investigators who were allowed for de-encryption were stored by a Trusted Third Party (ZorgTTP). The encryption code and the data entered into the app were sent to a research database and were only decrypted to review the physician-reported outcome. Data files used for analysis will be stored on a local safe network storage facility.

## 3 Results

Of all patients eligible for inclusion during the study period, 1019 patients were included (total hip arthroplasty (THA) 46 %, total knee arthroplasty (TKA) 54 %). Baseline and outcome characteristics are summarized in Table 2. During the first 2 postoperative weeks, the app was used by more than 80 % of patients per day (Fig. 1). The app use declined during the third and fourth week from 80 % to 30 %, consistent with the recommendation that use of the app beyond 2 weeks was only needed if new drainage or other complications would occur.

**Table 3:** Reported postoperative wound drainage in all patients with and without prosthetic joint infection.

	First week		Second week		Third week		Fourth week	
	No PJI	PJI	No PJI	PJI	No PJI	PJI	No PJI	PJI
App use per week ( n patients)	978	16	950	16	999	11	999	4
Wound drainage								
No wound drainage at all during week	416 (43 %)	5 (31 %)	789 (83 %)	2 (13 %)	903 (90 %)	4 (36 %)	973 (97 %)	3 (75 %)
Any wound drainage anywhere during week	489 (50 %)	10 (63 %)	115 (12 %)	14(88 %)	76 (8 %)	7 (64 %)	25 (3 %)	1 (25 %)
Minimal ( <2×2 cm on gauze)	424 (87 %)	8 (80 %)	98 (85 %)	12 (86 %)	65 (86 %)	3 (43 %)	24 (96 %)	1 (100 %)
Mild ( >2×2 cm on gauze)	181 (37 %)	7 (70 %)	25 (22 %)	5 (36 %)	19 (25 %)	3 (43 %)	4 (16 %)	1 (100 %)
Moderate (1–2 gauze swabs exchanged)	41 (8 %)	4 (40 %)	10 (9 %)	6 (43 %)	1 (1 %)	5 (71 %)	1 (4H %)	–
Heavy ( >2 gauze swabs exchanged)	10 (2 %)	–	2 (2 %)	2 (14 %)	–	–	–	–
New onset drainage after 1week no drainage	–	–	28 (5 %)	2 (13 %)	25 (5 %)	2 (50 %)	4 (1 %)	1 (25 %)
>4 d of wound drainage during week	82 (8 %)	3 (19 %)	31 (3 %)	4 (25 %)	11 (1.1 %)	1 (9 %)	4 (0,4 %)	1 (25 %)
Drainage not assessable ${}^{*}$	165 (17 %)	1 (6 %)	50 (5 %)	–	23 (2 %)	–	1 (0.1 %)	–
Redness								
Any wound redness during week	100 (10 %)	3 (19 %)	45 (5 %)	3 (19 %)	37 (4 %)	3 (19 %)	20 (2 %)	0 (0 %)
Increased redness	32 (32 %)	1 (33 %)	20 (44 %)	2 (66 %)	9 (24 %)	0 (0 %)	6 (30 %)	0 (0 %)
Fever								
Fever during postoperative period	53 (5 %)	–	21 (2 %)	1 (6 %)	12 (1 %)	2 (18 %)	5 (0,5 %)	1 (25 %)
Pain								
VAS >5 anytime during week	360 (37 %)	5 (33 %)	114 (12 %)	1 (7 %)	47 (5 %)	1 (13 %)	41 (4 %)	–
VAS >7 anytime during week	107 (11 %)	0 (0 %)	19 (2 %)	0 (0 %)	8 (0,8 %)	0 (0 %)	6 (0,6 %)	0 (0 %)
Alerts								
Any alerts during week	415 (42 %)	8 (53 %)	250 (26 %)	6 (40 %)	101 (10 %)	0 (0 %)	66 (7 %)	0 (0 %)

${}^{*}$ Patients with or without any drainage who could not assess wound drainage during 1 or more days during week due to gauze swabs in situ.

**Figure 2:**
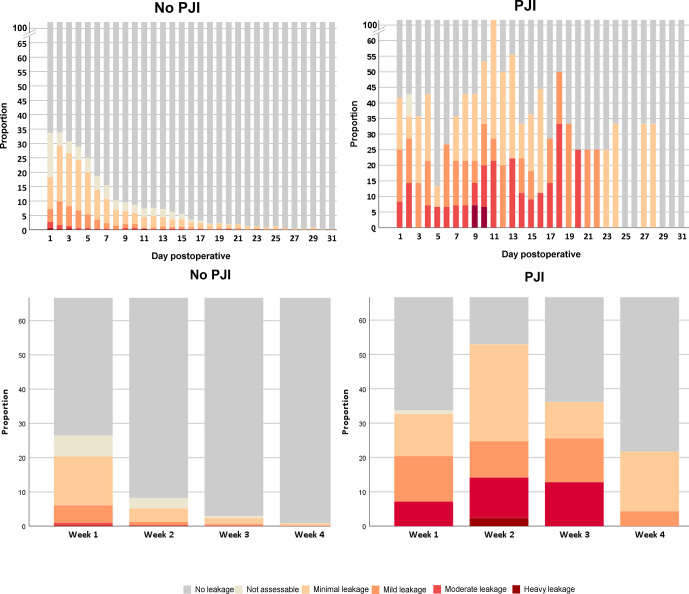
Reported extent and duration of postoperative wound drainage in patients with and without PJI.

The incidence of postoperative wound drainage in patients with and without PJI is reported in Table 3 and Fig. 2. During the first, second, third and fourth postoperative weeks, any form of wound drainage was present in 50 %, 12 %, 8 % and 3 % of patients without PJI and in 63 %, 88 %, 64 % and 25 % of patients with PJI. The high proportion of drainage in the first week was predominantly caused by minimal leakage (defined as <2×2 cm on gauze) occurring in 87 % ( 424/489 ) of patients without PJI. In this group, 51 patients (5 %) had moderate to heavy wound drainage in the first week, decreasing to 1 %, 1 % and 0.1 % in the next weeks. Moderate to heavy wound drainage of patients with PJI occurred in 25 %, 38 %, 46 % and 0 % of patients during 4 weeks. Reported redness (10 %), fever (5 %) and high pain scores (VAS >7 , 11 %) were mainly reported during the first week and declined thereafter. Proportions of wound drainage in patients without PJI varied depending on the type of joint, BMI and the presence of diabetes (Table 4).

**Table 4:**
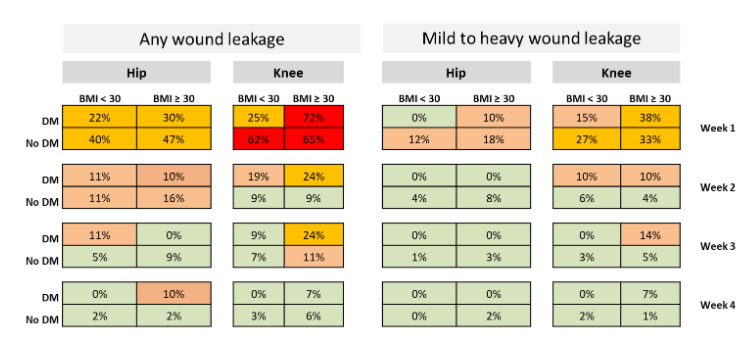
Percentage of postoperative wound drainage in 1003 patients without PJI during the postoperative course.

Legend: BMI – body mass index; DM – diabetes mellitus.

**Table 5:**
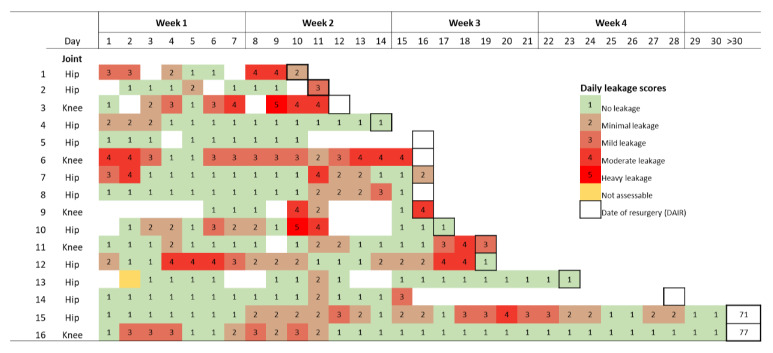
Drainage scores in 16 patients who developed a prosthetic joint infection.

On verification of the patient files, patient 5 appeared to have stopped using the app on day 11 and subsequently developed wound drainage, redness and pain in the next 3 d without recording it in the app. On day 15, this patient was admitted with PJI. For patient 14, no drainage data between day 14 and 28 could be retrieved from the patient notes.

Sixteen (1.6 %) patients developed a PJI during the follow-up period. Fourteen patients experienced an early postoperative PJI after a median of 14 d (IQR 10–18 d). Two patients developed an early chronic PJI on postoperative day 71 and day 77 (Table 5). Three patients were reoperated for a suspected PJI that was subsequently not confirmed (e.g. haematoma). Six patients (0.6 %) received a short course of antibiotics for a presumed superficial wound infection but did not develop a PJI. The strongest risk factors for PJIs were any wound drainage in the second week (OR 50.83, 95 % CI 11.41–226.51) or moderate to heavy drainage in the second (OR 51.22, 95 % CI 15.84–165.65) or third week (OR 103.23, 95 % CI 26.08–408.57). New onset drainage in the second week after a week without drainage (OR 80.71, 95 % CI 9.12–714.52) and more than 5 cumulative wound drainage days during the first 3 postoperative weeks (OR 9.20, 95 % CI 3.37–25.14) were also strongly associated with the development of PJI (Table 6). Drainage for more than 5 d during the first 3 weeks predicted PJI with sensitivity of 63 % and specificity of 87 %, while drainage for more than 10 d predicted PJI with sensitivity of 27 % and specificity of 97 % (Appendix B). No wound drainage whatsoever was reported by 467 patients (46 %). Of them, only one patient developed a PJI resulting in a negative predictive value of no wound drainage as indicator for recovery without PJI of >98% (Table 6). The positive predictive value of any amount of wound drainage for PJI was low during the 4 postoperative weeks (2 %, 11 %, 8 % and 4 %, respectively) and increased for moderate–heavy wound drainage, especially in the third postoperative week (8 %, 35 %, 83 %, 0 %, respectively). Over the 4-week postoperative period, the average number of alerts per patient was not higher for patients with PJI compared to patients without PJI (OR 1.37 (0.39–4.87). Of the 18 420 d of app use, an alert was sent 2589 (14 %) times to 498 patients. In total, 141 (6.6 %) annotations could be obtained from the electronic patient files confirming that patients had contacted the hospital based on the sent alert. This led to a change of treatment in 61 (43 %) patients as summarized in Appendix C. Of the 16 patients who developed a PJI, an alert was sent in the preceding period to six patients which resulted in earlier outpatient evaluation or hospital admission in three patients.

**Table 6:** Comparison of risk factors for failure in patients with and without PJI.

	No PJI	PJI	OR	Sens	Spec	PPV	NPV
	( n=1003 )	( n=16 )	(95 % CI)	(%)	(%)	(%)	(%)
Any drainage							
First week	489/978	10/16	1.67 (0.60–4.62)	63	50	2	99
Second week	115/950	14/16	50.83 (11.41–226.51)	88	88	11	100
Third week	76/999	7/11	21.25 (6.09–74.22)	64	92	8	100
Fourth week	25/999	1/4	12.99 (1.31–129.24)	25	97	4	100
Moderate–heavy drainage							
First week	47/978	4/16	6.60 (2.05–21.25)	25	95	8	99
Second week	11/950	6/16	51.22 (15.84–165.65)	38	99	35	99
Third week	1/999	5/11	103.23 (26.08–408.57)	45	100	83	99
Fourth week	1/999	0/4	–	0	100	0	100
New drainage after first week without drainage							
Second week	28/480	5/6	80.71 (9.12–714.52)	83	94	15	100
Third week	25/512	3/5	29.22 (4.67–182.85)	60	95	11	100
Fourth week	4/512	1/3	63.50 (4.74–850.04)	33	99	20	100
>5 cumulative leaking days during day 1–21							
Second to fourth week	123/1003	9/16	9.20 (3.37–25.14)	56	88	7	99
Moderate–heavy drainage and/or fever and/or redness							
First week	164/978	6/16	2.98 (1.07–8.31)	38	83	4	99
Second week	68/950	7/16	10.09 (3.65–27.92)	44	93	9	99
Third week	47/999	6/11	21.00 (6.21–70.99)	55	95	11	99
Fourth week	23/999	1/4	14.15 (1.42–141.17)	25	98	4	100
>2 alerts based on algorithm ${}^{*}$							
First week	141/978	3/16	1.37 (0.39–4.87)	19	86	2	98
Second week	128/950	1/16	0.43 (0.06–3.27)	6	87	1	98
Third week	69/999	0/11	–	0	93	0	99
Fourth week	47/999	0/4	–	0	95	0	100

Legend: PJI – prosthetic joint infection; OR – odd ratio; sens – sensitivity; spec – specificity; PPV – positive predicted value; NPV – negative predicted value. ${}^{*}$ Algorithm is defined in Appendix A.

## 4 Discussion

### 4.1 Principal findings

In the current study, a detailed overview of self-reported wound characteristics in the first month after arthroplasty while using a mobile wound care app provided important clinical insights. Complete absence of wound drainage during the first postoperative month was a sensitive and specific predictor of recovery without PJI. From the second week onward, wound drainage was strongly associated with the occurrence of PJI, but the positive predictive value remained low. Generation of an alert by the algorithm did not adequately identify patients with PJI.

### 4.2 Strengths and weaknesses

A major strength of this study is the unbiased prospective and daily information of exactly defined postoperative wound characteristics as provided by patients with an easy-to-use smartphone application. Another strength is the large number of included patients without PJI that enabled us to create infographics of uncomplicated wound drainage for several subgroups of patients (Table 4).

This study has several limitations. The COVID-19 pandemic led to a temporary suspension of inclusions between March and May 2020 and continued to have a huge impact on the number of inclusions in the following year. The mean age of study participants (62.7 years for THA and 64.6 years for TKA) in our cohort was lower than the reported mean age in the Dutch Arthroplasty Registry involving all arthroplasties in the Netherlands (69.9 years for THA and 68.4 years for TKA in 2021), indicating that elderly patients may have been less willing to use the app. The relatively low number of patients with PJI in the study may have had an impact on the outcome. An even larger study would increase the precision of the results. Remarkably, patients who developed a PJI reported a relatively low proportion of wound redness and fever. We hypothesize that some patients with PJI symptoms may have visited their orthopaedic surgeon without registering their symptoms in the app, but this remains speculative.

The short follow-up of at least 3 months is a limitation of this study in which we focused on the relation between wound drainage and early postoperative PJI. For late acute haematogenous PJI, initial wound drainage is probably not relevant because bacteremia is mostly the source of PJI. However, some patients with a chronic PJI will have been missed in our study and these patient may have had prolonged initial wound drainage providing a route for Coagulase-negative staphylococci to reach the implant and cause late chronic PJI. This would have resulted in an even stronger reported association between wound drainage and PJI than reported in this study. This needs to be further investigated in a follow-up study.

### 4.3 Implications of our findings

This study has three important implications. First, moderate to heavy wound drainage in the third week strongly predicted PJI with a number needed to operate to diagnose one PJI in 1.2 patients. Although this predictor was only derived from a small subset of patients with PJI, moderate to heavy drainage was nearly absent in patients without PJI. Therefore, these patients need urgent clinical assessment of the postoperative wound to decide whether the patient should be operated for a suspected PJI or not.

Second, persistent wound drainage and wound drainage in the second and third postoperative weeks was strongly associated with the development of PJI. However, positive predicted values were low due to the many patients with wound drainage during those weeks who did not develop PJI. From patients who had any form of drainage that was regarded as a suspected PJI during the second postoperative week, 10 patients would need to be operated to find one PJI. This indicates that, even with a strong association between drainage and PJI, wound drainage alone is not an accurate predictor for presence of PJI in this group. The strength of the association did not increase significantly when fever and wound redness were added to wound drainage as risk factors, which may relate to the earlier mentioned low proportion of these symptoms reported by patients.

Third, wound drainage in the first postoperative week was not indicative of PJI. The high proportion of reported wound drainage during this week (Table 3, Fig. 4) is explained by several factors: (1) drainage was recorded from the very first postoperative day (not from discharge from hospital), (2) minimal wound drainage could have occurred during only 1 day of this week to be counted as wound drainage and (3) drainage was minimal (defined as <2×2 cm on the gauze) in 87 % of the patients with drainage in the first week ( 424/489 patients). Only 5 % ( n=51 ) of patients in this group had moderate to heavy wound drainage. In the second postoperative week, wound drainage dropped down to 12 %, again with minimal drainage ( <2×2 cm on gauze) in most (85 %) of these patients.

This study confirmed that in patients without any wound drainage, an early postoperative PJI is very unlikely. With mobile health applications, this subgroup of patients can be easily identified during follow-ups and fewer outpatient visits may be needed during follow-ups which may reduce costs. The postoperative use of bandages during the first weeks to cover the postoperative wound may have resulted in underreporting of wound drainage. However, the impact was estimated to be similar in patients with and without PJI as the use of bandages was identical for all patients. We also assessed whether the closing technique (use of either glue or staples) was associated with the duration of postoperative wound drainage after hip arthroplasty during the first two weeks, which was not the case (staples 3.2 d, glue 2.9 d, p=0.52 ).

Only one out of the 16 patients with a PJI received more than two alerts prior to the PJI, indicating that the used algorithm was inadequate for predicting PJI. This may be explained by the low threshold in the algorithm for sending alerts secondary to pain and mild wound drainage. Many alerts were sent for minimal wound drainage or relatively mild pain scores not related to PJI. Unfortunately, a low number of alert-based treatment adaptations could be retrieved from the patient files, making evaluation of the alerts sent by the application speculative. Patients apparently made the right decision not to call their physician as no PJI occurred in 98 % of them. The predictive value of the algorithm may be improved by using a machine learning algorithm, making iterative changes when the number of data increases thus allowing an automated update of the algorithm. Adding parameters like an increase in C-reactive protein may also increase the yield of the algorithm. Further, based on the current study, no “at-risk” points should be given for minimal wound drainage and low pain scores.

## 5 Conclusions

Detailed knowledge of the extent and duration of wound drainage after arthroplasty is vital for orthopaedic surgeons who consider to reoperate patients with postoperative wound drainage for a suspected PJI. In this study, in which a mobile health application was used to monitor patients after arthroplasty, PJI was very unlikely in patients without any wound drainage. From the second week onward, wound drainage was strongly associated with the occurrence of PJI, but the sensitivity and positive predictive value of wound drainage as a single predictor for PJI was low. Due to the limited follow-up of 3 months, some patients with a late chronic PJI may have been missed. The insights from this study may help clinicians to evaluate postoperative patients who present with a leaking wound. Future research should focus on optimizing the algorithm, thereby improving the predictive value of the alert function.

## Figures and Tables

**Figure A1:**
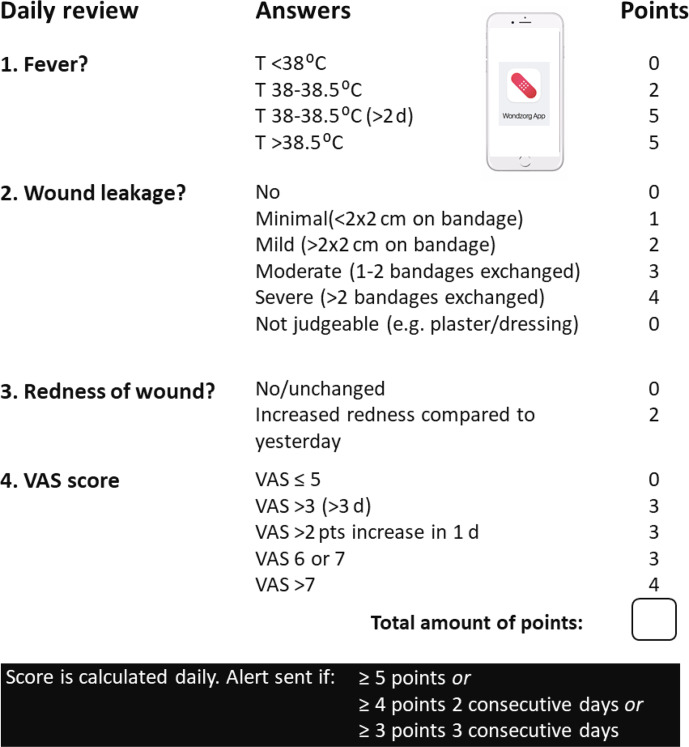
Calculated scores from the app and algorithm for sending alert to patients.

**Figure B1:**
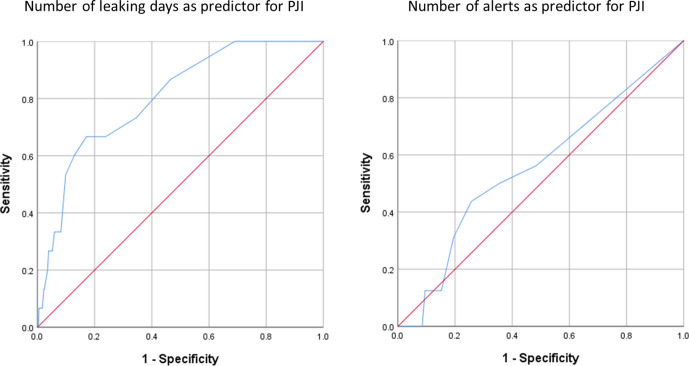
Receiver operating characteristic (ROC) curve using duration of leakage days or number of sent alerts as cutoff value for detecting PJI.

## Data Availability

Patient data were collected using an online database. The data are not publicly accessible but can be provided by the corresponding author, upon request.

## Appendix C

**Table C1:** Actions taken on algorithm-based alerts generated by the app from 18 437 daily reports.

	All	No PJI ( n=1013 )	PJI ( n=16 )
Total alert count	2590 (14 %)		
Alerts received ( n patients)	498/1019 (48.9 %)		
Alert first week postoperative ( n patients)	423/498 (84.9 %)		
Alert second week postoperative ( n patients)	232/498 (46.6 %)		
Alert third week postoperative ( n patients)	97/498 (19.5 %)		
Alert fourth week postoperative ( n patients)	68/498 (7.0 %)		
Alerts per individual patient (median)	3		
Reported patient–physician contact based on alerts	141/2124 (6.6 %) ${}^{a}$	135 (13 %)	6 (40 %)
Outcome of patient–physician contact			
No action needed	51 (36 %)	51	0
Adjust pain medication	34 (24 %)	34	0
Earlier outpatient evaluation	24 (17 %)	22	2
Admission to hospital	3 (2 %)	2	1
Other ${}^{b}$	29 (21 %)	26	3

${}^{a}$ From 466 alerts, patient files could not be checked for placed phone calls. ${}^{b}$ Practical wound management advice, Deep Venous Thrombosis excluded, patient not yet discharged.

## Team list

Wound care app study group: Enrike van der Linden (Leiden University Medical Centre, Leiden, the Netherlands), Demien Broekhuis (Leiden University Medical Centre, Leiden, the Netherlands), Menno Benard and Astrid Mol (Alrijne Hospital, Leiden, the Netherlands), Marijn Rutgers, Jantsje Pasma, Roos Bazuin and Nina Mathijssen (Reinier Haga Orthopaedic Centre, Zoetermeer, the Netherlands), Nienke Willigenburg and Myke van der Hoorn (Onze Lieve Vrouwe Gasthuis, Amsterdam, the Netherlands), Baukje Dijkstra (Medical Centre Leeuwarden, Leeuwarden, the Netherlands), Lex de Jong (Rijnstate Hospital, Arnhem, the Netherlands), Liesbeth Paans and Wouter Bekkers, Elisabeth TweeSteden Ziekenhuis, Henry Haan and Martin Stevens (University Medical Centre Groningen, Groningen, the Netherlands), Max Reijman (Erasmus Medical Centre Rotterdam, Rotterdam, the Netherlands), Carolien Kooijman and Hendrike Hoogeboom (Nij Smellinghe Hospital, Drachten, the Netherlands) Chris Meijer and Hieron Maree (Park Medical Centre, Rotterdam, the Netherlands), and all co-authors mentioned at the start of the paper.
